# Exosomes from mesenchymal stem/stromal cells: a new therapeutic paradigm

**DOI:** 10.1186/s40364-019-0159-x

**Published:** 2019-04-04

**Authors:** Kan Yin, Shihua Wang, Robert Chunhua Zhao

**Affiliations:** 1 Center of Excellence in Tissue Engineering, Department of cell biology, Institute of Basic Medical Sciences Chinese Academy of Medical Sciences, School of Basic Medicine Peking Union Medical College, Beijing, China; 2 Department of Biochemistry and Molecular biology, Basic medical college, Qingdao University, 308 Ningxia Road, Qingdao, 266071 China

**Keywords:** Exosomes, Mesenchymal stem cell, Clinical trial, Disease

## Abstract

Mesenchymal stem/stromal cells (MSCs) have been demonstrated to hold great potential for the treatment of several diseases. Their therapeutic effects are largely mediated by paracrine factors including exosomes, which are nanometer-sized membrane-bound vesicles with functions as mediators of cell-cell communication. MSC-derived exosomes contain cytokines and growth factors, signaling lipids, mRNAs, and regulatory miRNAs. Increasing evidence suggests that MSC-derived exosomes might represent a novel cell-free therapy with compelling advantages over parent MSCs such as no risk of tumor formation and lower immunogenicity. This paper reviews the characteristics of MSC exosomes and their fate after in vivo administration, and highlights the therapeutic potential of MSC-derived exosomes in liver, kidney, cardiovascular and neurological disease. Particularly, we summarize the recent clinical trials performed to evaluate the safety and efficacy of MSC exosomes. Overall, this paper provides a general overview of MSC-exosomes as a new cell-free therapeutic paradigm.

## Background

Mesenchymal stem/stromal cells (MSCs) are one of the most commonly employed cell types as a cell-based therapy for treating human diseases. Recently, several mechanisms have been put forward regarding the therapeutic potential of MSCs, including (1) paracrine factors involving proteins/peptides and hormones and (2) the transfer of exosomes/microvesicles packaging various molecules [1]. The therapeutic potential of mesenchymal stromal cells (MSCs) may be largely mediated by paracrine factors contained in vesicles [2]. Extracellular vesicles (EVs) from many cell sources have now been recognized as important messengers in intercellular communication via transfer of bioactive lipids, proteins, and RNAs. EVs are generally divided into 3 subgroups depending on their biogenesis; (a) exosomes, with a diameter of 40–150 nm, which are released into the extracellular when multivesicular bodies fuse with the cell membrane, (b) microvesicles, with a diameter of 150–1000 nm, originating from direct budding of the plasma membrane and finally (c) apoptotic bodies, which display a broad size distribution (50–2000 nm) [3]. Exosomes are crucial messengers that present in biological fluids and are involved in multiple physiological and pathological processes [4]. Today, there are hundreds of clinics and hundreds of clinical trials using human MSCs with very few, if any, focusing on the in vitro multipotential capacities of these cells, these cells home in on sites of injury or disease and secrete bioactive factors that are immunomodulatory and trophic (regenerative) [5]. One advantage of using exosomes is to get around MSCs’ side effects, exosomes are nanoparticles that can penetrate blood brain barrier and avoid potential pulmonary embolism related to transplantation of MSCs [6]. Knowledge of exosomes is essential to shed light on the functions of these vesicles on clinical applications. In this review, we focus on the mechanisms of exosomes covering the current knowledge on their potential cell-free therapeutic applications for MSC-derived exosomes.

### Exosomes

Exosomes are a family of nanoparticles with a diameter in the range of 40–150 nm that are generated inside multivesicular bodies (MVBs) and are secreted when these compartments fuse with the plasma membrane [7]. Upon the fusion of MVBs with the plasma membrane, exosomes are released into the extracellular and can be either taken up by target cells residing in the microenvironment or carried to distant sites via biological fluids [8]. Exosomes are enriched in many bioactive molecules such as lipids, proteins, mRNAs, transfer RNA (tRNA), long noncoding RNAs (lncRNAs), microRNAs (miRNAs) and mitochondrial DNA (mtDNA) [9]. Most exosomes have an evolutionarily conserved set of proteins including tetraspanins (CD81, CD63, and CD9), heat-shock proteins (HSP60, HSP70 and HSP90), ALIX and tumor susceptibility gene 101 (TSG101); however, they also have unique tissue type-specific proteins that reflect their cellular sources [10]. It has been reported that exosomes may be released from multiple cell types, including immunocytes [11], tumor cells [12], and mesenchymal stem/stromal cells (MSCs) [13]. Exosomes have received the most attention and have been implicated in physiological functions and in pathological conditions. Exosomes released by malignant cells play an important role in cancer cell communication with their microenvironment. HCC cell HepG2-derived exosomes could be actively internalized by adipocytes and caused significant transcriptomic alterations and in particular induced an inflammatory phenotype in adipocytes [14]. Exosomal miRNAs can affect many aspects of physiological and pathological conditions in HCC and indicates that miRNAs in exosomes can not only serve as sensitive biomarkers for cancer diagnostics and recurrence but can also potentially be used as therapeutics to target HCC progression [15].

#### Characteristics of MSC-derived exosomes

The abundance of cargos identified from MSC-derived exosomes function largely via the constant transfer of miRNAs and proteins, > 150 miRNAs [16] and > 850 unique protein [17] have been identified in the cargo of MSC-derived exosomes, resulting in the alteration of a variety of activities in target cells via different pathways. Many miRNAs have been found in MSC-derived exosomes and are reportedly involved in both physiological and pathological processes such as organism development, epigenetic regulation, immunoregulation (miR-155 and miR-146) [18], tumorigenesis and tumor progression (miR-23b, miR-451, miR-223, miR-24, miR-125b, miR-31, miR-214, and miR-122) [19]. Over 900 species of proteins have been collected from MSC-derived exosomes according to ExoCarta. Several studies have also shown that exosomes derived from MSCs harbor cytokines and growth factors, such as TGFβ1, interleukin-6 (IL-6), IL-10, and hepatocyte growth factor (HGF), which have been proven to contribute to immunoregulation [20]. Comparable levels of VEGF, extracellular matrix metalloproteinase inducer (EMMPRIN), and MMP-9 have been reported in MSC-derived exosomes, these three proteins play a vital role in stimulating angiogenesis, which could be fundamental for tissue repair [21].

#### The fate of injected MSC-derived exosomes

Current knowledge of the biodistribution of EVs upon administration in animal models is limited. Do MSC-derived exosomes have a favorable biodistribution and pharmacokinetic profile? Several strategies have been employed for in vivo tracking to determine EVs biodistribution upon systemic delivery in different animal models [22, 23]. Near-infrared (NIR) dyes are ideal for in vivo applications due to their high signal/noise ratio [24]. EVs with superparamagnetic iron oxide nanoparticles for high resolution and sensitive magnetic resonance analysis provide for accurate detection also in deep organs [25]. In an intracerebral hemorrhage rat model, DiI-labeled MSC-derived exosomes reached brain, liver, lung, and spleen after intravenous injection [26]. Exosomes appear to be able to home to the injury site. In the mouse model of acute kidney injury (AKI), DiD-labeled EVs were accumulated specifically in the kidneys of mice with AKI compared with healthy controls [27]. Intranasal administration led to better brain accumulation of exosomes at the injured brain site, compared to i.v. injection [28]. Biodistribution of systemically administered EVs is a dynamic process: a rapid phase of distribution in liver, spleen, and lungs within approximately 30 min upon administration is followed by an elimination phase via hepatic and renal processing, removing EVs in 1 to 6 h after administration [29].

### Therapeutic effects of MSC-derived exosomes

#### Liver diseases

The application of MSCs in animal models of liver fibrosis/cirrhosis and acute liver injury, eventually, in patients ameliorates the progress of the disease. Li et al. found that the exosomes derived from human umbilical cord MSCs (hucMSC) ameliorate liver fibrosis by inhibiting both the epithelial-mesenchymal transition of hepatocytes and collagen production, significantly restore the serum aspartate aminotransferase activity and inactivate the TGF-β1/Smad2 signaling pathway by decreasing collagen type I/III and TGF-β1 and the phosphorylation of Smad2 [30]. Tan et al. found that HuES9.E1 MSC-derived exosomes elicit hepatoprotective effects through an increase in hepatocyte proliferation, as demonstrated by high expression of proliferation proteins (proliferating cell nuclear antigen and Cyclin D1), the anti-apoptotic gene Bcl-xL and the signal transducer and activator of transcription 3 (STAT3) [31]. Liver regeneration was significantly stimulated by MSCs culture medium (MSC-CM) as shown by an increase in liver to body weight ratio and hepatocyte proliferation. MSC-CM upregulated hepatic gene expression of cytokines and growth factors relevant for cell proliferation, angiogenesis, and anti-inflammatory responses, treatment with MSC-derived factors can promote hepatocyte proliferation and regenerative responses in the early phase after surgical resection [32]. Transplantation of exosomes released from adipose derived-MSCs (AD-MSC) can significantly reduce the elevated serum levels of alanine aminotransferase and aspartate aminotransferase, liver inflammation and necrosis in concanavalin A (Con A)-induced hepatitis in C57BL/6 mice as well as the serum levels of proinflammatory cytokines, including tumor necrosis factor-α (TNF-α), interferon-γ (IFN-γ), IL-6, IL-18 and IL-1β, and the inflammasome activation in mouse liver [33].

#### Kidney disease

Mesenchymal stem/stromal cells (MSCs) have shown promising results in experimental acute kidney injury (AKI) and chronic kidney disease (CKD). Systemic administration of human umbilical cord-derived MSCs (huMSCs)-derived EVs in rats with renal Ischemia-reperfusion injury (IRI) increased renal capillary density and reduced fibrosis by direct transfer of the proangiogenic factor vascular endothelial growth factor (VEGF) and mRNAs involved in this process [34]. A single intrarenal administration of adipose tissue-derived autologous MSCs-derived EVs in pigs with renal artery stenosis attenuated renal inflammation, disclosed by decreased renal vein levels of several pro-inflammatory cytokines, including TNF-α, IL-6, and IL-1-β. Contrarily, renal vein levels of IL-10 increased in EV-treated pigs, associated with a shift from pro-inflammatory to reparative macrophages populating the stenotic kidney, underscoring the immunomodulatory potential of EVs [35]. Microvesicles derived from human bone marrow MSCs stimulated proliferation in vitro and conferred resistance of tubular epithelial cells to apoptosis. In vivo, microvesicles accelerated the morphologic and functional recovery of glycerol-induced acute kidney injury (AKI) in SCID mice by inducing proliferation of tubular cells. Microarray analysis and quantitative real time PCR of microvesicle-RNA extracts indicate that microvesicles shuttle a specific subset of cellular mRNA, such as mRNAs associated with the mesenchymal phenotype and with control of transcription, proliferation, and immunoregulation [36]. The effects of bone marrow MSCs-derived MVs in SCID mice survival in lethal cisplatin-induced acute renal injury (AKI) was to exert a pro-survival effect on renal cells in vitro and in vivo mainly ascribed to an anti-apoptotic effect of MVs. MVs up-regulated in cisplatin-treated human tubular epithelial cells anti-apoptotic genes, such as Bcl-xL, Bcl2 and BIRC8 and down-regulated genes that have a central role in the execution-phase of cell apoptosis such as Casp1, Casp8 and LTA [37]. Intravenous injection of EVs isolated from the conditioned medium of human umbilical cord MSCs after unilateral renal ischemia preserved kidney function and decreased serum levels of the AKI marker neutrophil gelatinase-associated lipocalin [38]. Human bone marrow MSCs-derived exosomes contain insulin-like growth factor-1 receptor (IGF-1R) mRNA. Exosomal transfer of IGF-1R mRNA to damaged renal tubular cells promoted their proliferation and repair and this effect was significantly reduced when IGF-1R transcription in donor cells was silenced [39].

#### Cardiovascular disease

There are preclinical studies in which MSC-derived exosomes are used for treating cardiovascular diseases (CVDs) such as AMI, stroke, pulmonary hypertension, and septic cardiomyopathy [40]. Cui et al. demonstrated adipose-derived MSC (AdMSC)-derived exosomes led to a markedly increase in cell viability of H9C2 cells under hypoxia/reoxygenation (H/R) in vitro, and administration of AdMSC-derived exosomes protected ischemic myocardium from myocardial ischemia-reperfusion (MI/R) injury via activation of Wnt/β-catenin signaling in vivo [41]. Furthermore, Wang et al. showed superior cardioprotective effects of endometrium-derived MSCs (EmMSC) in a rat myocardial infarction (MI) model as compared to BMSCs and AdMSCs. These differences may be caused by certain miRNAs particularly miR-21 enrichment in exosomes secreted from EmMSCs, which exerted effects on cell survival and angiogenesis by targeting PTEN [42]. HuES9.E1 derived MSCs-derived exosomes treatment increased levels of ATP and NADH, decreased oxidative stress, increased phosphorylated-Akt and phosphorylated-GSK-3β, reduced phosphorylated-c-JNK in ischemic/reperfused hearts to enhance myocardial viability and prevented adverse remodeling after myocardial ischemia/reperfusion injury [43]. Feng et al. determined that miR-22 is highly enriched in exosomes secreted by mouse bone marrow-derived MSCs after ischemic preconditioning, and administration of these exosomes significantly reduced infarct size and cardiac fibrosis by targeting methyl-CpG-binding protein 2 (Mecp2) in a mouse myocardial infarction (MI) model [44]. Both bone marrow MSCs and their derived exosomes are cardioprotective against myocardial infarction in animal models. However, anti-miR-125b treatment of exosomes significantly attenuated their protective effect [45]. MiR-21-5p plays a key role in hMSC-exo–mediated effects on cardiac contractility and calcium handling, likely via PI3K signaling [46]. In a rat myocardial ischaemia reperfusion injury model, injection of bone marrow-derived MSCs-derived exosomes reduced apoptosis and myocardial infarct size and subsequently improved heart functions by inducing cardiomyocyte autophagy via AMPK/mTOR and Akt/mTOR pathways [47].

#### Neurological disease

MSC-Exosomes have shown potential therapeutic benefit in the treatment of neurological and neurodegenerative diseases. One of the most outstanding results in the field is the fact that systemically injected exosomes are able to cross the blood-brain barrier (BBB) and achieve the brain parenchyma. Systemic delivery of targeted exosomes containing a siRNA against α-synuclein reduced the mRNA and protein levels of α-synuclein in the brain [48, 49]. Xin et al. also reported that rat bone marrow derived MSCs derived EVs enriched with the miR-17-92 cluster enhanced oligodendrogenesis neurogenesis neural plasticity and functional recovery after stroke possibly by suppressing PTEN and subsequently by increasing the phosphorylation of proteins downstream of PTEN including of the protein kinase B/mechanistic target of rapamycin/glycogen synthase kinase 3β signaling pathway [50]. Katsuda et al. used exosomes secreted from human adipose tissue-derived MSCs that contain large amounts of neprilysin, the most prominent enzyme that degrades β-amyloid peptide in the brain. Transfer of exosomes into neuroblastoma N2a cells led to reductions in both secreted and intracellular β-amyloid peptide levels, which might be a therapeutic approach to Alzheimer’s disease [51]. The results of migration assay and capillary network formation assay showed that exosomes secreted by adipose-derived stem cells (ADSCs-Exos) promoted the mobility and angiogenesis of brain microvascular endothelial cells (BMECs) after oxygen-glucose deprivation (OGD) via miR-181b-5p/TRPM7 axis [52]. Injection of exosomes from mouse bone marrow MSCs could rescue cognition and memory impairment according to results of the Morris water maze test, reduced plaque deposition, and Aβ levels in the brain; could decrease the activation of astrocytes and microglia; could down-regulate proinflammatory cytokines (TNF-α and IL-1β); and could up-regulate anti-inflammatory cytokines (IL-4 and -10) in AD mice, as well as reduce the activation of signal transducer and activator of transcription 3 (STAT3) and NF-κB in APP/PS1 double transgenic mice [53].

##### Immune disease

**Fig. 1:**
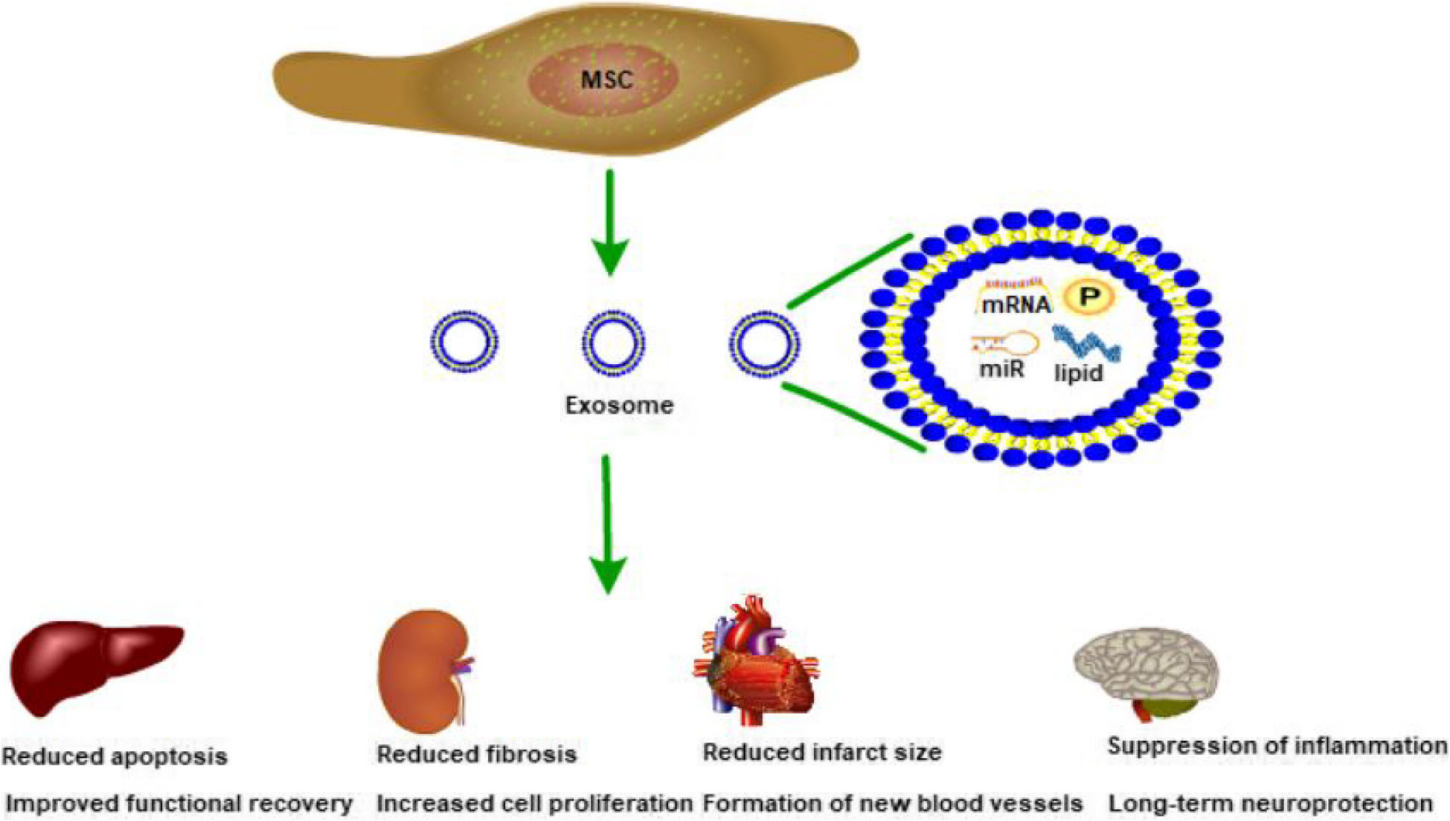
Therapeutic effects of MSC-derived exosomes. Exosomes from MSCs contain multiple proteins, lipids, RNAs (mRNA, miRNA, ncRNA). Therapeutic effects of MSC-derived exosomes in liver, kidney, cardiovascular, and neurological diseases

Potent immunomodulatory properties of MSCs-exo has been evaluated. Exosomes have been observed to play crucial roles in carrying and presenting functional MHC-peptide complexes to modulate tumor-specific T cell activation [54]. Exosomes released from Bone marrow (BM)-derived MSCs can effectively ameliorate chronic graft-versus-host disease (cGVHD) in mice by inhibiting the activation and infiltration of CD4 T cells, reducing pro-inflammatory cytokine production, as well as improving the generation of IL-10-expressing Treg and inhibiting Th17 cells [55]. Human multipotent stromal cells-derived EVs suppress autoimmunity in models of type 1 diabetes (T1D) and experimental autoimmune uveoretinitis (EAU). EVs inhibit activation of antigen-presenting cells and suppress development of T helper 1 (Th1) and Th17 cells, they also increased expression of the immunosuppressive cytokine IL-10 and suppressed Th17 cell development [56]. Human bone-marrow derived MSCs exosomes promote Tregs proliferation and immunosuppression capacity by upregulating suppressive cytokines IL-10 and TGF-β1 in PBMCs of asthmatic patient [57]. MiR-181c in human umbilical cord MSCs-derived exosomes is key to anti-inflammatory effects in burned rat inflammation model by downregulating the TLR4 signaling pathway [58] Fig. 1.

### Clinical trials of MSCs exosomes–based therapies

The use of MSC-derived EVs for regenerative therapy requires the production and isolation of a suitable quantity of clinical grade EVs from cultured MSCs [59]. While complexities surrounding the therapeutic potential of MSCs exosomes continue to unravel, several clinical trials (Table 1, data from http://clinicaltrials.gov) have been completed or are underway in order to evaluate this therapeutic potential. Among them, determining the optimal dose, the appropriate time window for exosome administration and route of administration that achieves maximal efficacy without adverse effects are the most important issues to resolve [60]. Improved preclinical study quality in terms of treatment allocation reporting, randomization and blinding will accelerate needed progress towards clinical trials that should assess the feasibility and safety of this therapeutic approach in humans [61]. For example, MSC-exosomes will be great biological tools for cancer therapy, it is hopeful to delve deeper into the potential of MSC-exosomes among cancer cells and provide effective treatments with the highest safety [62] Table 2.

**Table 1:** The function of MSC-derived exosomes

Source of Exosomes	Specific Disease Treated	Target/Pathway	Reference
human umbilical cord MSCs	liver fibrosis	TGF-β1/Smad2	[30]
HuES9.E1 MSC	hepatoprotective effects	Cyclin D1, Bcl-xL, STAT3	[31]
adipose derived-MSCs	hepatitis	TNF-α, IFN-γ, IL-6, IL-18 and IL-1β	[33]
human umbilical cord-derived MSCs	renal Ischemia-reperfusion injury (IRI)	VEGF	[34]
adipose tissue-derived autologous MSCs	renal artery stenosis	TNF-α, IL-6, IL10 and IL-1-β	[35]
human bone marrow MSCs	acute kidney injury	mRNAs	[36]
bone marrow MSCs	acute renal injury	Bcl-xL,Bcl2, BIRC8,Casp1, Casp8 and LTA	[37]
human umbilical cord MSCs	unilateral renal ischemia	lipocalin	[38]
bone marrow MSCs	acute kidney injury	mRNAs	[36]
Human bone marrow MSCs	damaged renal tubular	IGF-1R	[39]
adipose-derived MSC	myocardial ischemia-reperfusion injury	Wnt/β-catenin	[41]
endometrium-derived MSCs	myocardial infarction	miR-21, PTEN	[42]
HuES9.E1 derived MSCs	myocardial ischemia/reperfusion injury	PI3K/Akt	[43]
mouse bone marrow-derived MSCs	myocardial infarction	miR-22, Mecp2	[44]
bone marrow MSCs	myocardial infarction	miR-125b	[45]
human mesenchymal stem cell	cardiac contractility	miR-21-5p, PI3K	[46]
bone marrow-derived MSCs	myocardial ischaemia reperfusion injury	AMPK/mTOR, Akt/mTOR	[47]
rat bone marrow derived MSCs	stroke	miR-17-92, PTEN	[50]
human adipose tissue-derived MSCs	Alzheimer’s disease	neprilysin	[51]
adipose-derived stem cells	oxygen-glucose deprivation	MicroRNA-181b/TRPM7	[52]
mouse bone marrow MSCs	Alzheimer’s disease	STAT3, NF-κB	[53]
bone marrow derived MSCs	chronic graft-versus-host disease	Treg, Th17	[55]
human multipotent stromal cells	type 1 diabetes, uveoretinitis	Th1, Th17	[56]
human bone-marrow derived MSCs	asthma	IL-10, TGF-β1	[57]
human umbilical cord MSCs	inflammation	MiR-181c, TLR4	[58]

**Table 2:** Clinical trials of MSCs exosomes–based therapies

Study title	Disease	Intervention	Phase	NCT
Allogenic Mesenchymal Stem Cell-Derived Exosome in Patients With Acute Ischemic Stroke	Cerebrovascular Disorders	Biological: exosome	Phase 1 Phase 2	NCT03384433
MSC-Exos Promote Healing of MHs	Macular Holes	Biological: exosomes derived from mesenchymal stem cells (MSC-Exo)	Early Phase 1	NCT03437759
microRNAs Role in Pre-eclampsia Diagnosis	Preeclampsia	Biological: exosome	Complete	NCT03562715
Effect of Microvesicles and Exosomes Therapy on β-cell Mass in Type I Diabetes Mellitus (T1DM)	Diabetes Mellitus Type 1	Biological: MSC exosomes.	Phase 2 Phase 3	NCT02138331
Trial of a Vaccination With Tumor Antigen-loaded Dendritic Cell-derived Exosomes	Non Small Cell Lung Cancer	Biological: Dex2	Phase 2	NCT01159288
Serum Exosomal Long Noncoding RNAs as Biomarkers for Lung Cancer Diagnosis	Lung Cancer (Diagnosis)	Diagnostic Test: collect samples		NCT03830619

## Conclusions

MSCs most exert their therapeutic effects through the secretion of factors to reduce cellular injury and enhance repair. MSC exosomes probably function in a similar fashion, namely as a communication vehicle secreted by MSCs to affect the stromal support functions through the maintenance of a dynamic and homeostatic tissue microenvironment [63]. MSC exosomes may have the versatility and capacity to interact with multiple cell types within the immediate vicinity and remote areas to elicit appropriate cellular responses. MSCs through their secreted exosomes target housekeeping processes to restore tissue homeostasis and enable cells within the tissue to recover, repair and regenerate. This hypothesis provides a rationale for the therapeutic efficacy of MSCs and their secreted exosomes in a wide spectrum of diseases and rationalizes the additional use of MSC exosomes as an adjuvant to support and complement other therapeutic modalities [64]. Nonetheless, the exact mechanism of in vivo action of exogenously administered exosomes, their biodistribution, pharmacokinetics, and possibility of targeted delivery are not fully elucidated. New techniques may help in filling this gap of knowledge and further promoting clinical translation of exosomes-based regenerative therapy [65].

## Abbreviations

AKI: Acute kidney injury
BBB: Blood-brain barrier
CKD: Chronic kidney disease
EVs: Extracellular vesicles
H/R: Hypoxia/reoxygenation
MI/R: Myocardial ischemia-reperfusion
MSCs: Mesenchymal stem/stromal cells
MVBs: multivesicular bodies
NIR: Near-infrared
OGD: Oxygen-glucose deprivation
